# Cytoreductive Surgery Plus Hyperthermic Perioperative Chemotherapy for Selected Patients with Peritoneal Metastases from Colorectal Cancer: A New Standard of Care or an Experimental Approach?

**DOI:** 10.1155/2012/309417

**Published:** 2012-07-19

**Authors:** Paul H. Sugarbaker

**Affiliations:** Washington Cancer Institute, Program in Peritoneal Surface Malignancy, Washington, DC 20010, USA

## Abstract

Peritoneal metastases (PM) are a common presentation for patients with metastatic colorectal cancer (CRC), and the median survival of patients with PM is approximately one year. In a majority of patients, the disease remains limited to the peritoneal cavity. Therefore, investigators have applied cytoreductive surgery (CRS) and heated perioperative chemotherapy (HIPEC) as a standard approach for selected patients with PM from CRC. These investigators have demonstrated a very promising long-term survival in a subset of patients with a limited amount of isolated peritoneal metastatic disease. This paper presents the data that supports CRS and HIPEC as a treatment option for CRC patients with PM. These results of treatment are compared and contrasted to the results that can be expected with systemic chemotherapy alone.

## 1. Introduction

As new responsibilities are recognized by the surgeon, he/she must expand the science of surgical technology in order to bring about progress in patient care. In the surgical management of PM the major innovation in the craft of surgery is the peritonectomy procedure. Removal of large areas of peritoneum involved by cancer implants is an important new surgical technology. Also, the use of chemotherapy in the operating room or in the early postoperative period is a surgical innovation specifically used for the management of PM and peritoneal mesothelioma. When used as a combined treatment, CRS (including peritonectomy) and HIPEC have become a treatment option repeatedly reported with favorable results for patients with PM, from colorectal malignancy. As occurs with many other diseases, knowledgeable patient selection is an important aspect of surgical management. This paper will critically evaluate the evidence that supports this treatment strategy for PM and make recommendations to optimize the management of the peritoneal surface component of colorectal cancer dissemination.

## 2. Cytoreductive Surgery and Hyperthermic Perioperative Chemotherapy as a Treatment Option

The efficacy of CRS and HIPEC as a treatment option for PM from CRC is an established part of the oncologic literature. The survival benefits were well described before the use of modern treatments for metastatic colorectal cancer, using oxaliplatin, irinotecan, and molecular agents. In 1995, three-year survival of 35% in 51 patients with PM from colon cancer treated only with CRS plus intraperitoneal 5-fluorouracil and mitomycin C was shown [1]. In 2003, Verwaal and coworkers in Amsterdam published a three-year projected survival of 38% in 54 patients treated by CRS and hyperthermic intraperitoneal mitomycin C with adjuvant systemic 5-fluorouracil [2]. Shen and coworkers, between 1991 and 2002, treated 77 nonappendiceal CRC patients with CRS and HIPEC. They concluded that one-third of patients with complete resection have long-term survival and that systemic chemotherapy did not contribute to the control of PM in these patients [3]. These studies, performed in the absence of modern CRC chemotherapy agents, document the efficacy of CRS and perioperative chemotherapy to rescue approximately one-third of these patients.

Some medical oncologists question the benefits of CRS and HIPEC now that oxaliplatin, irinotecan, and molecular agents are available. Are the benefits of systemic chemotherapy alone so great that CRS plus HIPEC are not needed? Or is the best option, at our current state of knowledge, a multidisciplinary approach using the best surgical and best medical oncology treatments available? Franko and colleagues presented data to show that these two strategies were most effective when combined. They showed that the median survival was longer in patients treated by modern systemic chemotherapy when CRS and HIPEC were added to the clinical pathway [4]. Until more data becomes available, patients with PM from CRC have the right to be informed of a possible curative treatment option and it is the oncologists' obligation to provide the relevant information in a timely manner.

### 2.1. Cytoreductive Surgery and Hyperthermic Perioperative Chemotherapy for Peritoneal Metastases Provides an Individualized Treatment Strategy

Currently, the medical oncologic standard of care for stage IV CRC involves treatments that are nearly identical for all patients. A routine surgery is followed by a routine systemic chemotherapy regimen. A routine followup by physical examination and CT then occurs. If symptoms or radiologic findings suggest metastases, a palliative surgery may be performed, followed by second line chemotherapy. This current standard of care fails to recognize that metastatic disease is a complex process and that the anatomic sites of treatment failure vary greatly between individuals. In those patients who have a high risk for local-regional failure, or are determined to have PM at the time of cancer recurrence, a treatment specifically directed at the abdominal and pelvic surfaces is appropriate.

### 2.2. A New Strategy for Colorectal Peritoneal Metastases

The new strategy demands a surgical procedure combined with cancer chemotherapy in the operating room. Cytoreductive surgery involves five different peritonectomy procedures that are combined as needed with eight different visceral resections, in order to make patients with PM visibly disease-free [6]. Immediately following the complete cancer resection and prior to intestinal reconstruction, the abdominal and pelvic spaces are flooded by a warm chemotherapy solution with agents augmented by heat. These treatments may be continued with early postoperative intraperitoneal chemotherapy (EPIC), using cell cycle-specific drugs that should contact all visceral and parietal surfaces because their use precedes the development of abdominal adhesions [7]. 

#### 2.2.1. Quantitative Prognostic Indicators

**Figure 1:**
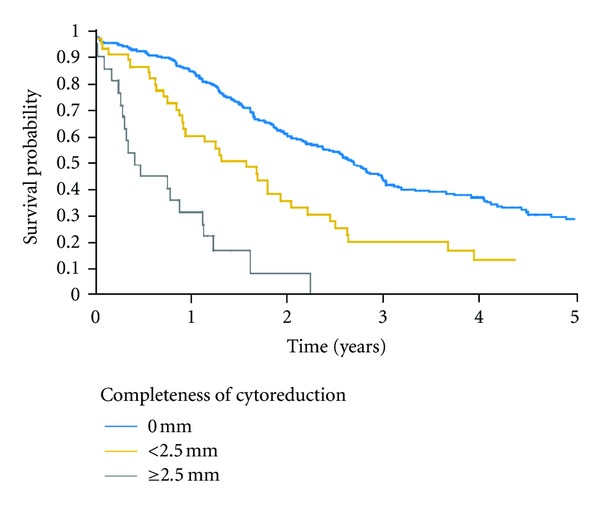
Prognostic impact of the completeness of cytoreductive surgery (*P* < 0.001) on overall survival [5].

As with nearly all successful surgical interventions, the proper selection of patients for treatment is an important requirement for long-term benefit. It is well documented that the results of CRS and HIPEC for PM vary greatly with the clinical status of the patient being treated. Quantitative prognostic indicators have been established that allow the cancer surgeon to predict the likelihood of long-term benefit [8]. An important prognostic indicator is the completeness of cytoreduction (CC) score; for CRC, a complete cytoreduction indicates that the surgeon was successful in clearing all visible sites of disease (CC-0), or he left behind only a few minute deposits of cancer that are expected to be eradicated by the HIPEC (CC-1). Complete cytoreduction includes both CC-0 and CC-1 CRS. A necessary requirement for long-term benefit in the management of PM is complete cytoreduction. A French multicentric trial that studied 523 patients compares survival with complete versus incomplete cytoreduction. The data is shown in Figure 1 [5].

**Figure 2:**
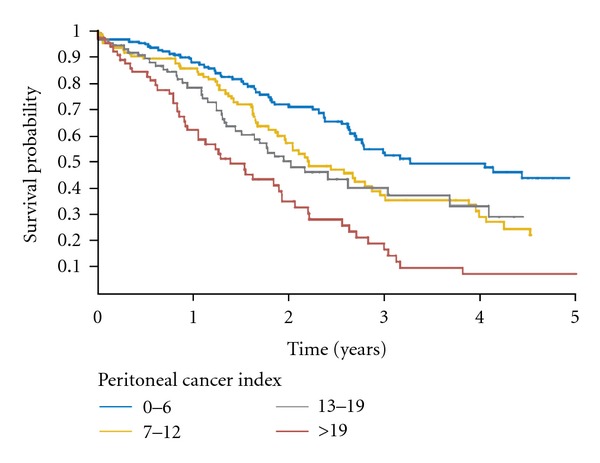
Prognostic impact of the extent of carcinomatosis (i.e., peritoneal cancer index; *P* < 0.001) on overall survival [5].

A second useful quantitative prognostic indicator is the peritoneal cancer index (PCI). This prognostic indicator scores both the distribution and extent of PM in 13 abdominal and pelvic regions to arrive at a quantitative assessment of the extent of disease [8]. The PCI predicts the likelihood of an incomplete cytoreduction; it also predicts long-term survival even if the cytoreduction is complete [9]. A low PCI indicating a limited extent of carcinomatosis is associated with an improved prognosis. Elias and coworkers in the French collaborative study of 523 patients reported a 50% survival at 5 years with a PCI of 6 or less, 27% 5-year survival with PCI between 7 and 19, and less than 10% with PCI greater than 19 [5]. Elias suggested that the very extensive CRS required in patients who have a PCI of greater than 20 should only occur under special circumstances such as a very young and fit patient. These data showing the impact of PCI on survival are shown in Figure 2.

#### 2.2.2. Extent of Disease as a Major Determinant of Prognosis with Peritoneal Metastases

The concept of increasing benefits of treatment with reduced extent of disease may be a valid concept throughout oncology. It is the basic hypothesis that drives the TNM system. Certainly, it operates in the treatment of CRC liver metastases. The greater the number of deposits resected from the liver, the poorer the prognosis; this is true even though an R0 liver resection is achieved. It should not be surprising that this concept of increasing benefits with lesser extent of disease is important for interpreting the results of treatment of CRC PM. 

The two prognostic indicators, CC score and PCI, taken together tell us that the extent of PM has a profound effect on treatment outcome when this manifestation of metastatic disease is treated by CRS and HIPEC. A reliable concept worthy of pursuit states that the likelihood of long-term survival will continue to improve as extent of disease at the time of definitive treatment decreases. Clinically, this means that definitive treatment early in the progression of PM can be expected to optimize the survival benefit. 

Support for this concept of early intervention with small volume of PM can be found in the recent report by Elias and colleagues [10]. They describe a new plan for early intervention in patients at high risk for local-regional and peritoneal surface progression after primary CRC surgery. These patients had small volume peritoneal seeding, ovarian metastases, or perforation through the primary cancer at the time of colon resection. After treatment with systemic chemotherapy, the patients were taken back to the operating room for a “systematic second-look surgery.” Return for reoperation was within one year. At exploration, Elias and colleagues found cancer present in 16 of the 29 patients (55%). All patients in whom progressive disease was found were treated with CRS and HIPEC. At 27 months, median followup for the survival of these patients, was 50%. The high incidence of prolonged survival in this group of patients with PM undergoing early definitive second-look surgery, supports the concept of maximal benefit in patients treated with a minimal burden of disease.

#### 2.2.3. Proactive Intervention in Patients with Colorectal Peritoneal Metastases

An important clinical question concerns the general application of this concept of proactive intervention in the management of CRC PM. Currently, CRS and HIPEC may not be considered a reasonable treatment option by the medical oncologist because the risks of treatment are perceived to exceed its benefit. The perception is that these patients must submit to a high-risk major surgical intervention with an extended hospitalization and long convalescence. Unfortunately, the alternative to comprehensive management, using CRS and HIPEC, is death from disease progression. Within a few months or perhaps a few years, the PM will progress. These patients now with high volume intra-abdominal disease have no treatment options except palliative surgery. This provides a minimum short-term benefit, if any benefit at all. Perhaps valid in the past, this nihilistic attitude toward the management of PM needs to change. Late referral by the medical oncologist after all systemic chemotherapy options have failed and the patients are now symptomatic from the PM should be considered a part of oncologic history. The proactive management of every patient with PM needs to be included in the discussions within the multidisciplinary team. 

#### 2.2.4. As Morbidity and Mortality Decreases the Referral for Definitive Treatment Expands

It is no doubt that three decades of clinical and laboratory studies in PM have allowed the surgical team to ascend a learning curve that results in increased benefit and reduced toxicity. This learning curve is a phenomenon repeatedly observed with a complex surgical procedure and it has been reported with CRS and HIPEC [11, 12]. As the adverse events decrease, the reluctance of the medical oncologist to refer a patient for definitive treatment of PM should disappear. In our last 150 cytoreductions at the Washington Cancer Institute, 60% of patients had 3, 4, or 5 peritonectomy procedures. Eighty-five percent had 2 or more visceral resections, and 50% had at least one anastomosis. These patients with colorectal or appendiceal PM had a major surgical intervention with an average time in the operating room of 9.6 hours. Despite this extensive CRS with HIPEC, there was a single postoperative mortality (0.6%) and a serious complication rate of 12% [13]. Numerous publications regarding the current expectation for adverse events associated with CRS and HIPEC have been recently published [14]. 

### 2.3. Absence of Evidence-Based Medicine Regarding Management of Peritoneal Metastases from Colorectal Cancer by Systemic Chemotherapy Alone

There is no doubt that modern systemic chemotherapy using oxaliplatin, irinotecan, and maximal doses of fluorouracil have led to a prolongation of life in patients with metastatic CRC. The molecular agents, bevacizumab and cetuximab, has also had a modest effect on survival [15]. However, in the multiple publications regarding the medical oncologic management of metastatic disease, data regarding the objective response rate, progression-free survival, and survival in the subset of patients with PM have not been made available. Data regarding systemic chemotherapy treatment of a general population of patients with CRC metastases is not at all relevant to PM patients. Unfortunately, reliable data from the medical oncologic literature regarding the survival of patients with CRC PM does not exist.

#### 2.3.1. Comparatively Poor Survival of Patients with Peritoneal Metastases

Patients with PM should not be assumed to have the same benefits as those patients with liver metastases or lung metastases who have indicator lesions by radiologic tests and, therefore, are included in the data regarding survival with modern chemotherapy regimens. Patients with PM have a more limited survival when compared to patients with liver or other systemic metastases if systemic chemotherapy is the only treatment [16]. Franko and coworkers compared the survival of 364 patients with PM from CRC to 1731 patients who had CRC metastases in the absence of PM. The median overall survival was reduced to 12.7 months in patients with PM as compared to 17.6 months in patients without PM (HR 1.3 (95% CI 1.2–1.5)). The anatomic location of liver metastases, lung metastases, and retroperitoneal lymph node metastases is such that disease progression can occur in many patients without substantial disruption of function. In contrast, PM, even of small size, will soon result in bowel obstruction, fistulization, or bowel perforation. Palliative surgical interventions in this clinical situation are of short-term benefit. The bowel is a delicate organ whose essential nutritional function can be completely disrupted even by a small volume of PM. 

#### 2.3.2. Fundamental Differences in the Progression of Metastases to Parenchymal Organs as Compared to Peritoneal Surface Metastases

**Figure 3:**
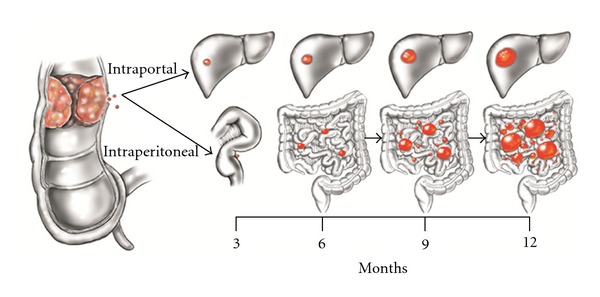
Schematic diagram that presents a theoretical model comparing the progression of one colorectal liver metastasis to one peritoneal metastases over one year. The liver metastasis will expand within the liver parenchyma with a doubling time of approximately three months. The peritoneal metastasis will progress at approximately the same speed but will also exfoliate cancer cells into the free peritoneal space. Many cancer nodules of many different sizes will occur widely distributed throughout the abdomen and pelvis within one year.

Not only do PM, because of their intimate relationship with the bowel, rapidly disrupt host function, but also their progression is usually more rapid than with parenchymal metastases. The epithelial cell is programmed to exfoliate from its attachment to the basement membrane as the cancer nodule enlarges. Cancer nodules on peritoneal surfaces may exfoliate cancer cells in great numbers into the free peritoneal space (reviewed in [17]). Growth inhibition of these newly implanted cells does not exist and nodules rapidly progress, and then cause additional peritoneal implants. The exponential progression of disease to multiple sites on the abdominal and pelvic peritoneal surfaces may rapidly cause the death of the patient. Without surgical interventions, PM will progress much more rapidly than metastases at other anatomic sites (Figure 3). 

#### 2.3.3. Site-Specific Reporting of Benefit for Treatment of Metastatic Disease Needed

The survival statistics in patients with metastatic disease at isolated anatomic sites should be reported separately. Brain metastases, lung metastases, liver metastases, retroperitoneal nodal metastases, and PM are likely to represent a strong biological variance of the process currently and collectively referred to as disseminated colorectal malignancy. Currently, all anatomic sites of disease are treated in the same manner using systemic chemotherapy. However, their responses will vary greatly with the anatomic location of the malignancy. Unfortunately, data regarding this difference in response and in survival does not exist in the oncologic literature. The clinician knows that the survival following detection of liver metastases, peritoneal, or other sites of metastases varies greatly. For example, brain metastases, of only a few centimeters in diameter, may disrupt function of the host completely within a few weeks of detection. The same size lesion in the liver or lung parenchyma may be totally asymptomatic and remain asymptomatic for years. Also, survival of disease at different anatomic sites will vary greatly because definitive surgical intervention may or may not be possible. Without CRS and HIPEC, PM of small size may have a profound effect on the host by disrupting enteral nutrition because of the intimate anatomic relationship of the cancer with the small bowel.

#### 2.3.4. Absence of Radiologic Monitoring of Peritoneal Metastases

It is not surprising that a firm assessment of the benefits of modern systemic chemotherapy on the survival of patients with PM has not been forthcoming. Large multi-institutional studies require an indicator lesion that can accurately assess changes in disease status over time. In patients with PM the radiologic assessment of disease progression is difficult—in most patients it is impossible. Only patients who have a large burden of PM and, therefore, a very limited survival are candidates for longitudinal radiologic followup. Usually these patients with limited survival expectations are not eligible for clinical trials with systemic chemotherapy.

The best data regarding survival of patients with PM comes from surgical studies of patients with isolated and possibly resectable disease. Elias and coworkers studied 48 patients with PM treated with palliative chemotherapy using modern chemotherapy agents [18]. The patients were not treated with CRS and HIPEC because this treatment modality was not available at their institution. The median survival was 23.9 months. In 48 matched patients, who had CRS and HIPEC, the median survival was 62.7 months with a statistically significant difference in survival (*P* < 0.5). Elias and coworkers concluded that chemotherapy alone using modern agents may achieve a median survival of 24 months and a 13% 5-year survival. However, CRS plus HIPEC resulted in a median survival of 63 months with a 51% 5-year survival. There are possible criticisms of these data presented by Elias and coworkers. The control group was treated by systemic chemotherapy alone, whereas the experimental group had second-look CRS plus HIPEC. It is possible that the control group, given the benefit of CRS, would have an improved survival in the absence of HIPEC.

Franko and colleagues studied 38 patients with PM who were matched to 67 patients who had CRS and HIPEC [4]. The median survival of patients receiving systemic chemotherapy was 16.8 months, whereas the survival of those treated by systemic chemotherapy plus CRS and HIPEC was 34.7 months. These differences were significant with a *P*-value of less than 0.001. Franko and colleagues concluded that CRS and HIPEC complement systemic chemotherapy for the management of PM; they should not, in a multidisciplinary approach, be considered to be competing therapies.

### 2.4. Evidence-Based Medicine Regarding Management of Peritoneal Metastases from Colorectal Cancer by Cytoreductive Surgery and Hyperthermic Perioperative Chemotherapy

In sharp contrast to the absence of data with systemic chemotherapy, evidence-based medicine regarding management of PM from CRC using CRS and HIPEC is extensive. Most relevant is the phase III study reported by Verwaal and colleagues in 2003 [2]. This landmark study compared 105 patients who were randomly assigned to receive either standard treatment with systemic 5-fluorouracil-leucovorin as compared to an aggressive CRS and HIPEC with mitomycin C. The patients in the “experimental therapy” also had systemic 5-fluorouracil chemotherapy. After a median followup of 21.6 months, the median survival was 12.6 months with systemic chemotherapy and 22.3 months with CRS and HIPEC (*P* = 0.032). These authors reported that a complete cytoreduction and a limited extent of disease were important determinants of benefit. The durability of the CRS and HIPEC was confirmed in a follow-up manuscript in 2008 [19].

From the perspective of a medical oncologist, a major criticism of the Verwaal phase III trial is the absence of modern systemic chemotherapy in the treatment of the control group. Would FOLFOX chemotherapy improve the survival of the control group to equal that of patients having CRS and HIPEC? Or, as might be expected, would modern systemic chemotherapy improve the survival of both groups of patients in this trial? It may be expected that CRS plus HIPEC plus FOLFOX are the optimal strategy for CRC PM [4].

Another important evidence-based report in support of these combined treatments was the multi-institutional registry of 506 patients reported by Glehen et al. in 2004 [20]. Glehen collected data on 506 patients from 28 institutions who had CRS and HIPEC for CRC PM. The overall median survival was 19.2 months but in patients in whom cytoreduction was complete, the median survival was 32.4 months; the survival was 8.4 months in patients in whom complete cytoreduction was not possible (*P* < 0.001). 

Yan and colleagues published a systematic review [21] of 64 publications from single institutions that reported results of this combined treatment modality. The systematic review contained two randomized controlled trials, one nonrandomized comparative study, and 11 observational studies without control groups, including one large multi-institutional study. Yan and colleagues concluded that the current evidence suggests that CRS combined with HIPEC is associated with improved survival as compared to systemic chemotherapy in patients with PM from CRC. 

The multi-institutional French study reported on 523 patients from 23 French-speaking centers [5]. The overall median survival was 30.1 months and 5-year overall survival was 27%. In 84% of patients who had a complete cytoreduction, the median survival was 33 months. This group concluded that CRS and HIPEC are now considered the standard of care for selected patients in the French guidelines for management of PM. 

Verwaal reported long-term Dutch multicenter data [22]. The survival of 562 patients at 10 years was 37%. From the extensive data of references 2, 4, 5, and 19–22, one must conclude that patients who had a CC-0 or CC-1 CRS had a median survival of 30–60 months and 5-year survival between 20 and 40%. 

Manuscripts that show a prolonged median survival and a definite 5-year survival benefit with CRS and HIPEC are being published on a regular basis in the oncology literature. These patients may be selected in that the favorable results only occur in patients who undergo complete macroscopic CRS. It is likely that this results in a selection bias compared to patients that only qualify for palliative systemic chemotherapy. Possible differences between these groups of patients need to be addressed in future studies. Nevertheless, similar reports showing the benefit of systemic chemotherapy for CRC PM are not being published. By default, CRS and HIPEC must be considered as a valid treatment option to be presented to selected patients with PM. Patients deserve to be informed.

#### 2.4.1. Long Followup (5 and 10 Years) Necessary to Evaluate Treatments

It is crucial to note the large difference in long-term survival produced by modern systemic chemotherapy as compared to long-term survival produced by CRS and HIPEC. Yes, both cause a prolongation in the median survival; this is estimated at approximately 20 months for systemic chemotherapy in contrast to 40 months for CRS and HIPEC. However, the great difference is the 30–40% of patients who show 5- and 10-year survival with the combined treatment. Such percentages of 5- and 10-year survival do not exist as part of the medical oncologic literature. The percent of long-term survival with CRS and HIPEC will depend on the experience of the institution, the PCI and CC scores of patients treated [5]. The best results will be realized when the multidisciplinary team considers all CRC PM patients for proactive treatment as an initial treatment option.

### 2.5. Peritoneal Metastases from Colorectal Cancer Compared to Peritoneal Metastases from Other Gastrointestinal Malignancies

CRS and HIPEC are not only of benefit to patients with CRC PM, but also for other gastrointestinal malignancies with peritoneal dissemination. As a general rule, it seems that the lower the biological aggressiveness of the malignancy, the greater the efficacy of CRS and HIPEC. For example, Sugarbaker showed a 65% survival of patients with pseudomyxoma peritonei (peritoneal metastases from appendiceal malignancy) who have a PCI greater than 20 [23]. This survival is present after 20 years of followup. This is in contrast to a 5-year survival of 7% in CRC patients with a PCI of 20 or greater [5].

**Figure 4:**
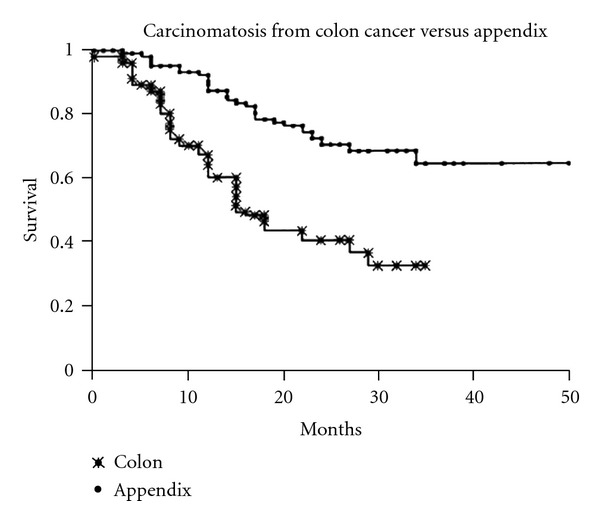
Comparison of survival of colon and appendiceal peritoneal metastases in patients having the same treatments [1].

Data comparing survival of patients with PM from CRC with patients who have appendiceal cancer is shown in Figure 4. There is no debate about the general application of CRS and HIPEC for patients with appendiceal mucinous neoplasms [1, 24].

Although CRS and HIPEC have produced some limited prolongation of survival in patients with gastric cancer, long-term survival in terms of a “cure” is rarely achieved [25, 26]. In gastric cancer, the important role for HIPEC may be in the adjuvant treatment of patients at high risk for local-regional and peritoneal dissemination [27]. It is largely ineffective in the treatment of documented PM, except in patients with a very low PCI.

### 2.6. Peritoneal Metastases from Colorectal Cancer Compared to Liver Metastases from Colorectal Cancer

Multicenter randomized controlled trials showing the benefit of the surgical removal of colorectal liver metastases as compared to nonoperative management have never been performed [28]. The survival of patients with resected liver metastases has often been compared to the survival of patients who have the combined treatment of PM [29, 30]. These results with peritoneal and liver metastases can also be compared to results of treatment with lung metastases [31]. By historical analogy and many precedents, the management of PM by CRS and HIPEC has been accepted and is being performed at most major institutions in the USA. 

### 2.7. Introducing Perioperative Chemotherapy into the Management of Primary Colorectal Cancer

If a patient with primary CRC and a small volume of PM or a patient with primary CRC at high risk for local-regional recurrence undergoes colon or rectal cancer resection at an institution that delivers perioperative chemotherapy, definitive treatment for peritoneal dissemination should be considered as a part of management of primary CRC. Pestieau and Sugarbaker reported on 5 patients with PM, in which the diagnosis was made at the time of primary CRC resection [32]. These patients had small volume of PM not evident by clinical signs or radiologic studies prior to colon resection. All patients survived at least 5 years. Two of these patients manifested late recurrences and eventually died of systemic disease. When a small volume of PM was definitively treated along with the resection of the primary CRC the outcome was favorable.

### 2.8. Requirements for Further Investigations

#### 2.8.1. Is HIPEC Essential in All Patients?

The combined treatment of CRS and HIPEC has been in use for over three decades for colorectal PM, and has consistently shown benefits in terms of long-term survival in a selected group of patients. Nevertheless, many important questions require an answer to optimize this treatment strategy. A basic question, as yet not clarified, concerns the contribution of the colorectal cancer to the CRS. Can some patients survive PM using complete CRS in the absence of HIPEC? If so, how can these patients be identified? In France, a randomized and controlled multi-institutional study to answer this question is currently accruing patients (Federation Nationale des Centres de Lutte Contre le Cancer: Cytoreductive Surgery with or without HIPEC for Colorectal Carcinomatosis). Thus, an answer to this question utilizing perioperative chemotherapy with 5-fluorouracil, oxaliplatin, and irinotecan should become available within several years. 

#### 2.8.2. Perioperative Chemotherapy Capable of Maintaining the Surgical Complete Response

After a surgical complete response has been achieved by CRS, the disease-free status within the abdomen and pelvis needs to be preserved. The most effective choice of combination chemotherapy delivered by both the intravenous and intraperitoneal routes, has not been determined in a clinical trial. Pharmacologic studies have suggested multiagent chemotherapy but further clinical studies to demonstrate the durability of the surgical complete response by HIPEC are indicated [33].

Another important issue is the optimal balance of chemotherapy effect and adverse events. Increasing the dose intensity and the number of perioperative chemotherapy agents is expected to increase the response; however, it is likely to result in increased toxicity. 

#### 2.8.3. Systemic Chemotherapy with Cytoreductive Surgery and Perioperative Chemotherapy

One aspect of treatment of PM that is well supported is the adjuvant use of systemic chemotherapy [4, 5, 20]. What has not yet been determined is the proper sequencing of the standard systemic treatments for metastatic disease with CRS and HIPEC. Some data exists to suggest that full dose neoadjuvant chemotherapy is associated with a reduced survival [20]. It is possible that full dose systemic chemotherapy caused a long delay definitive treatment that allowed for progression of peritoneal metastases in nonresponding patients. Also, full dose neoadjuvant chemotherapy may reduce the effectiveness of perioperative chemotherapy by causing acquired drug resistance. Bone marrow damage caused by a full 6 months of systemic chemotherapy requires a dose reduction of HIPEC and consequently a decreased dose intensity within the peritoneal cavity. This may translate into reduced cancer control within the peritoneal space. At this point in time, it is safe to say that the role of neoadjuvant FOLFOX chemotherapy in the management of PM has not been determined. The favorable results demonstrated in other gastrointestinal cancers need to be investigated for CRC PM.

Finally, the proper role for 5-fluorouracil (5-FU) in HIPEC has not been determined. A majority of successful chemotherapy regimens for gastrointestinal cancer are combinations of drugs added on to 5-FU at approximately 2000 mg/m^2^ per treatment cycle. This requirement for 5-FU has not been met in most HIPEC regimens. One possible plan to fulfill the need for 5-FU is to use intravenous 5-FU with HIPEC and then administer an additional 4 doses of EPIC 5-FU on postoperative days 1–4. If each 5-FU dose is 400 mg/m^2^, a 2000 mg/m^2^ dose has been achieved over 5 perioperative days.

## 3. Conclusion

Because of a large amount of mature data gathered over 30 years, one can begin to formulate the advantages and disadvantages associated with CRS and HIPEC for CRC PM (Table 1). The most important advantages is the long-term survival of 20–50% of a highly selected group of patients on surgical series. Secondly, the degree of PM as measured by CC and PCI scores allows selection of individuals who are most likely to benefit. 

There are disadvantages that remain. The procedure is associated with significant morbidity. Mortality from the operation appears to be less than 5% in centers of excellence and may be less than 1% in some centers. There is lack of uniformity of the patients that are entered into surgical databases, and lack of uniformity in the technical aspects of CRS and HIPEC treatments between the centers that do this procedure.

## Figures and Tables

**Table 1:** Cytoreductive surgery (CRS) and hyperthermic perioperative chemotherapy (HIPEC) for colorectal peritoneal metastases.

Credits	Debits
(i) Long-term survival in 30% of patients (ii) Patients with minimal carcinomatosis experience best survival	(i) The cytoreductive surgical procedure is complex and requires an extended learning curve
(iii) Morbidity (12%) and mortality (1%) at experienced centers is possible	(ii) Surgical series contain patients who have received many different HIPEC regimens at many different timepoints in their treatment
	(iii) The relative roles of CRS and HIPEC in the causation of long-term survival have not been determined
	(iv) Seventy percent of patients in the literature went on to die of peritoneal metastases usually because HIPEC did not sustain the surgical complete response
